# A novel technique for non-invasive assessment of pulmonary artery pressure in patients after the Fontan procedure

**DOI:** 10.1093/ehjopen/oeaf094

**Published:** 2025-08-05

**Authors:** David Backhoff, Hannah Quante, Marius Seitz, Carl Friedrich Wippermann, Christian Jux

**Affiliations:** Pediatric Heart Center, Department of Pediatric Cardiology, Intensive Care Medicine and Congenital Heart Disease, Justus-Liebig University Giessen, Feulgenstr. 10 – 12, Giessen 35392, Germany; Pediatric Heart Center, Department of Pediatric Cardiology, Intensive Care Medicine and Congenital Heart Disease, Justus-Liebig University Giessen, Feulgenstr. 10 – 12, Giessen 35392, Germany; Pediatric Heart Center, Department of Pediatric Cardiology, Intensive Care Medicine and Congenital Heart Disease, Justus-Liebig University Giessen, Feulgenstr. 10 – 12, Giessen 35392, Germany; Kinderarztpraxis Walluf, Hohlweg 20, Walluf 65396, Germany; Pediatric Heart Center, Department of Pediatric Cardiology, Intensive Care Medicine and Congenital Heart Disease, Justus-Liebig University Giessen, Feulgenstr. 10 – 12, Giessen 35392, Germany

## Introduction

In the ‘Fontan circulation’, the caval veins are directly connected to the pulmonary arteries.^1^ Due to absence of a subpulmonary ventricle, blood flow through the lungs significantly depends on pulmonary vascular resistance and pulmonary artery pressure (PAP) has an important impact on the outcome of those patients.^2^ The degree of venous congestion is reflected by PAP and central venous pressure (CVP) which are equal in the Fontan physiology^3^ in the supine but not in the upright position. Evaluation of PAP is of utmost importance as a value > 16 mmHg is considered a predictor of long-term mortality.^4^ However, measurement of PAP is an invasive procedure and thus performed at long intervals only. Therefore, we aimed to analyse the accuracy of a non-invasive method to assess PAP in subjects with Fontan circulation.

## Methods

For this prospective study, non-invasive PAP measurements were performed between 01/2024 and 02/2025 at the Pediatric Heart Center Giessen (Germany). We included children and young adults with a total cavopulmonary connection scheduled for cardiac catheterization. Patients with failing Fontan syndrome were excluded from this study.

**Figure 1:**
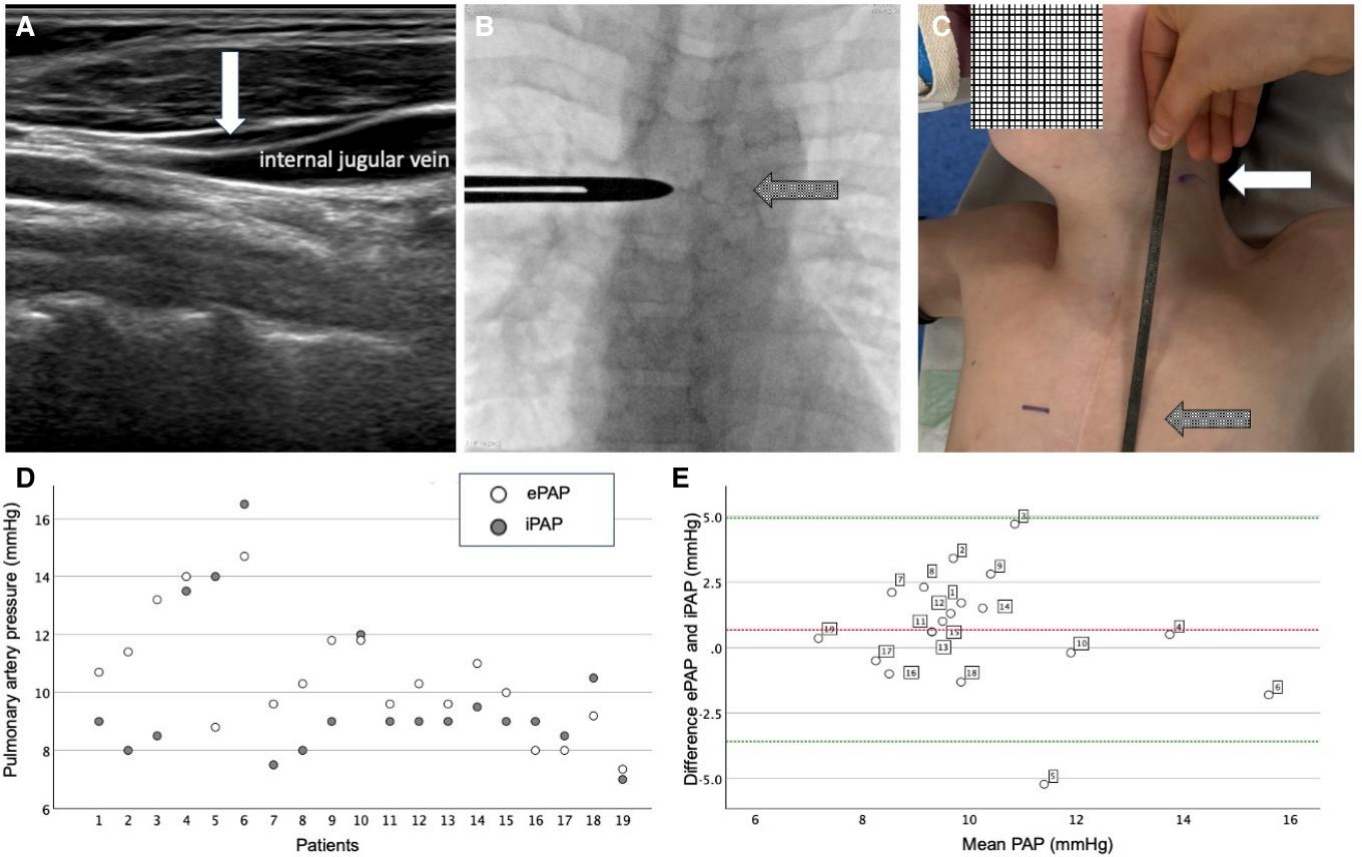
(*A*) The collapse point of the internal jugular vein was localized by ultrasound (white arrow) in an upright position and was marked on the skin. (*B*) At the end of catheterization, the level of the pulmonary arteries (PAs) was located by fluoroscopy and was also marked on the skin (grey arrow). (*C*) The craniocaudal distance between the collapse point of the internal jugular vein and the level of the PAs was measured and used for the estimation of PAP. (*D*) Estimated non-invasive pulmonary artery pressure (ePAP, open circles) and invasive measurements (iPAP, grey circles) of each participant. Note that ePAP and iPAP did not differ > 2 mmHg during the last 10 measurements. (*E*) Bland–Altman plot depicting agreement between ePAP and iPAP. The central red dotted line represents the mean difference (0.67 mmHg) between the iPAP and ePAP measurements while the green dotted lines represent the standard deviation. There was only one outlier (Subject No. 5).

First, the subjects sat at the edge of the catheterization table in an upright position. Using a linear transducer, the collapse point of the internal jugular vein was located by ultrasound (L12-5 50, Philipps, Netherlands, *Figure 1A*) and was marked on the skin. Subsequently, cardiac catheterization was performed in a lying position under sedation with propofol. Invasive PAP (iPAP) at both PAs was recorded. At the end of the procedure, the position of the PAs was marked on the chest under fluoroscopy guidance of and the craniocaudal distance between the PAs and the skin mark of the collapse point of the internal jugular vein was measured (*Figure 1B&C*). This distance was considered the height of the water column over the PAs and the estimated PAP (ePAP) was calculated as: ePAP (mmHg) = Distance (cm) × 0.74.

Statistical analysis was conducted using SPSS 29 (IBM SPSS Statistics, USA) and Bland–Altman plots were created.

## Results

Nineteen [6/19(32%) female] patients were included in this study. Median age was 17.9 (IQR 11.6–25.8) years. A total of 63% of the subjects (12/19) had a right systemic ventricle. Indication for cardiac catheterization was routine dilatation of already implanted stents in 13/19 (68%) subjects and cyanosis with the objective to identify and occlude veno-venous collaterals in 6/19 (32%) patients.

All patients had a preserved ventricular function, and median B-type natriuretic peptide concentration was 16 (IQR 9–49) pg/mL. On catheterization, median arterial oxygen saturation was 93 (IQR 91–95) %. Median iPAP was 9 (IQR 8.5–10.5) mmHg while ePAP was 10.3 (IQR 9.2–11.8) mmHg. Estimated PAP exceeded iPAP in 13/19 (68%) measurements. *Figure 1D* depicts these results in a Bland–Altman plot. Through the course of the study, the differences between iPAP and ePAP decreased, with a difference < 2 mmHg in the last 10 patients.

## Discussion

The present study demonstrates that non-invasive measurement of PAP by localization of the collapse point of the internal jugular vein using ultrasound is feasible in young subjects following the Fontan procedure. Pulmonary artery pressure plays an important role in the follow-up of patients after the Fontan operation as it reflects the degree of venous congestion before the ‘bottleneck’ of the univentricular physiology which is the blood flow through the lungs.^2^ A high PAP is associated with secondary organ dysfunction like Fontan-associated liver disease or protein-losing enteropathy and is an independent predictor of long-term mortality.^4^ However, measurement of iPAP is resource-intensive, time-consuming, carries risks such as infection, thrombosis, and vascular injury, and requires sedation, particularly in paediatric patients. Efforts have been made to find alternative methods to assess PAP in Fontan patients like measurement of peripheral venous pressure,^5^ ultrasound assessment of the hepatic vein flow pattern,^6^ and diameter of the inferior caval vein or biomarkers^7^ which remain either still invasive or inaccurate.

The technique described in this study is based on the location of the collapse point of the internal jugular vein by ultrasound, and has previously been demonstrated in adult subjects with biventricular physiology to asses CVP.^8,9^ However, our patients were seated upright instead of a 30° position like in healthy controls.^8^ Adjusting the examination position ensures that even elevated PAP can be measured in Fontan patients, as CVP in healthy individuals is normally lower.

The accuracy of this technique was evaluated by comparing non-invasively measured PAP with catheterization data. We found that ePAP was higher than iPAP in 13/19 subjects which is probably caused by the sedation during the measurement of iPAP leading to lower values. The patient position (sitting at ePAP vs. lying at iPAP assessment) is another important confounder as venous pooling and preload were higher in the horizonal position. Another finding of our study is that the accuracy of ePAP improved with increasing experience leading to PAP differences < 2 mmHg during the last 10 measurements. This improved accuracy was reached by measuring the air-line craniocaudal distance at the ventral aspect of the lying patient despite on the skin surface which avoids miscalculations due to scoliosis (like in Patient No. 5). It is of notice that this technique might be limited to normal or moderately elevated PAP as the sonographic window is limited at the cranial site by the lower edge of the mandible, especially in patients with short status. However, if the collapse point of the jugular vein could not be localized within the sonographic window, a high PAP should be assumed.

Another important factor is the assessment of the PA-level, which served as the ‘lower point’ of the water column in our patients. In this study, the level of the PAs was localized by fluoroscopy which is not feasible in an outpatient setting. Alternatively, the level of the PAs could be located by counting the dorsal ribs as the PAs are commonly situated at the level between the T5 and T6 vertebrae.^10^

Overall, non-invasive measurement of PAP provides information about the real-life PAP and appears to be a practical, accurate, and safer alternative to invasive methods in this unique patient population.

## Data Availability

Anonymized data of this study are available from the corresponding author upon reasonable request.

## Lead author biography

David Backhoff studied medicine at Justus-Liebig University of Giessen and Goethe University of Frankfurt. He trained in Pediatrics at the University Children's Hospital Göttingen, followed by fellowships and board certification in Pediatric Cardiology and Pediatric Intensive Care. Since 2020, he has been a consultant cardiologist at the Department of Pediatric Cardiology, Intensive Care Medicine and Congenital Heart Disease at Justus-Liebig University of Giessen. His clinical and research interests focus on interventional cardiology and electrophysiology in congenital heart disease.

## References

[1] Fontan F, Baudet E. Surgical repair of tricuspid atresia. Thorax 1971;26:240–248. 5089489 10.1136/thx.26.3.240 PMC1019078

[2] Gewillig M . The Fontan circulation. Heart 2005;91:839–846. 15894794 10.1136/hrt.2004.051789 PMC1768934

[3] Snarr BS, Paridon SM, Rychik J, Goldberg DJ. Pulmonary vasodilator therapy in the failing Fontan circulation: rationale and efficacy. Cardiol Young 2015;25:1489–1492. 26675595 10.1017/S1047951115002309

[4] Inai K, Inuzuka R, Ono H, Nii M, Ohtsuki S, Kurita Y, Takeda A, Hirono K, Takei K, Yasukouchi S, Yoshikawa T, Furutani Y, Shimada E, Shinohara T, Shinozaki T, Matsuyama Y, Senzaki H, Nakanishi T. Predictors of long-term mortality among perioperative survivors of Fontan operation. Eur Heart J 2022;43:2373–2384. 34888643 10.1093/eurheartj/ehab826

[5] Colman K, Alsaied T, Lubert A, Rossiter HB, Mays WA, Powell AW, Knecht S, Poe D, Ollberding N, Gao Z, Chin C, Veldtman GR. Peripheral venous pressure changes during exercise are associated with adverse Fontan outcomes. Heart 2021;107:983–988. 33127650 10.1136/heartjnl-2020-317179

[6] Franzon NH, Krzesinski LDS, Lintz VC, Ferraz IS, Damiano AP, Nogueira RJN, De Souza TH. Hepatic vein Doppler ultrasound to estimate central venous pressure in mechanically ventilated children. Eur J Pediatr 2024;183:5139–5147. 39325217 10.1007/s00431-024-05792-0

[7] Oka T, Kato R, Fumino S, Toiyama K, Yamagishi M, Itoi T, Hamaoka K. Noninvasive estimation of central venous pressure after Fontan procedure using biochemical markers and abdominal echography. J Thorac Cardiovasc Surg 2013;146:153–157. 23062410 10.1016/j.jtcvs.2012.09.021

[8] Xing CY, Liu YL, Zhao ML, Yang RJ, Duan YY, Zhang LH, Sun XD, Yuan LJ, Cao TS. New method for noninvasive quantification of central venous pressure by ultrasound. Circ Cardiovasc Imaging 2015;8:e003085. 25904575 10.1161/CIRCIMAGING.114.003085

[9] Wang L, Harrison J, Dranow E, Aliyev N, Khor L. Accuracy of ultrasound jugular venous pressure height in predicting central venous congestion. Ann Intern Med 2022;175:344–351. 34958600 10.7326/M21-2781

[10] Badshah M, Soames R, Khan MJ, Ibrahim M, Khan A. Revisiting thoracic surface anatomy in an adult population: a computed tomography evaluation of vertebral level. Clin Anat 2017;30:227–236. 27935171 10.1002/ca.22817

